# Unveiling microbiome changes in Mediterranean octocorals during the 2022 marine heatwaves: quantifying key bacterial symbionts and potential pathogens

**DOI:** 10.1186/s40168-023-01711-x

**Published:** 2023-12-05

**Authors:** Camille Prioux, Romie Tignat-Perrier, Ophélie Gervais, Tristan Estaque, Quentin Schull, Stéphanie Reynaud, Eric Béraud, Bastien Mérigot, Anaïs Beauvieux, Maria-Isabelle Marcus, Justine Richaume, Olivier Bianchimani, Adrien Cheminée, Denis Allemand, Christine Ferrier-Pagès

**Affiliations:** 1 Collège Doctoral, Sorbonne Université, Paris, France; 2 Unité de Recherche sur la Biologie des Coraux Précieux CSM - CHANEL, Centre Scientifique de Monaco, 8 Quai Antoine 1er, Monaco, MC-98000 Monaco Principality of Monaco; 3 Centre Scientifique de Monaco, 8 Quai Antoine 1er, Monaco, MC 98000 Principality of Monaco; 4 Septentrion Environnement, Campus Nature Provence, Marseille, 13008 France; 5 MARBEC, Univ. Montpellier, CNRS, IFREMER, IRD, Sète, France

**Keywords:** *Corallium rubrum*, *Paramuricea clavata*, Marine heatwaves, Climate change, Bacterial communities, Holobiont, *Endozoicomonas*, *Spirochaetaceae*, Gene expression, *16S rRNA* gene sequencing, qPCR

## Abstract

**Background:**

Climate change has accelerated the occurrence and severity of heatwaves in the Mediterranean Sea and poses a significant threat to the octocoral species that form the foundation of marine animal forests (MAFs). As coral health intricately relies on the symbiotic relationships established between corals and microbial communities, our goal was to gain a deeper understanding of the role of bacteria in the observed tissue loss of key octocoral species following the unprecedented heatwaves in 2022.

**Results:**

Using amplicon sequencing and taxon-specific qPCR analyses, we unexpectedly found that the absolute abundance of the major bacterial symbionts, *Spirochaetaceae* (*C. rubrum*) and *Endozoicomonas* (*P. clavata*), remained, in most cases, unchanged between colonies with 0% and 90% tissue loss. These results suggest that the impairment of coral health was not due to the loss of the main bacterial symbionts. However, we observed a significant increase in the total abundance of bacterial opportunists, including putative pathogens such as *Vibrio*, which was not evident when only their relative abundance was considered. In addition, there was no clear relation between bacterial symbiont loss and the intensity of thermal stress, suggesting that factors other than temperature may have influenced the differential response of octocoral microbiomes at different sampling sites.

**Conclusions:**

Our results indicate that tissue loss in octocorals is not directly caused by the decline of the main bacterial symbionts but by the proliferation of opportunistic and pathogenic bacteria. Our findings thus underscore the significance of considering both relative and absolute quantification approaches when evaluating the impact of stressors on coral microbiome as the relative quantification does not accurately depict the actual changes in the microbiome. Consequently, this research enhances our comprehension of the intricate interplay between host organisms, their microbiomes, and environmental stressors, while offering valuable insights into the ecological implications of heatwaves on marine animal forests.

Video Abstract

**Supplementary Information:**

The online version contains supplementary material available at 10.1186/s40168-023-01711-x.

## Background

Marine heatwaves (MHWs), characterized by episodes of prolonged abnormally high sea surface temperatures (SST), have increased in frequency, intensity and length in the world’s oceans in recent decades, fueled by global warming [19, 59]. In particular, the Mediterranean Sea is turning into one of the fastest-warming seas on Earth, with temperatures rising 20% faster than the global average [14, 52, 66] and a high occurrence of MHWs over the past two decades [18, 19, 39, 60]. In 2022, between three and seven record-breaking MHWs hit the Mediterranean Sea between May and September, with SST anomalies locally reaching 4.3 °C above the summer average, exceeding the previous maximum of +2–3 °C during the 2003 MHWs [33, 73, 92]. Recurrent MHWs have had devastating effects on community structure and health of marine animal forests (MAFs) [11, 32], which are among the most biodiverse assemblages in the Mediterranean Sea [61, 65, 81].

MAFs are unique habitats and important hotspots of biodiversity [9, 71, 78], creating macro- and microhabitats that provide shelter for a wide variety of marine species [69]. They also provide a range of valuable benefits to local populations, from fisheries to coastal protection and tourism [65, 81, 103]. Shallow and upper-mesophotic Mediterranean MAFs (< 60 m deep) are mainly composed of sponges and octocoral species including the gorgonians *Eunicella cavolini* (Koch, 1887), *Eunicella singularis* (Esper, 1791) and *Paramuricea clavata* (Risso, 1826), as well as the red coral *Corallium rubrum* (Linnaeus, 1758). Particularly vulnerable to disturbances, octocorals are being highly affected by MHWs that have caused outbreaks of microbial diseases and mortality episodes, which can affect up to 90% of the colonies in certain shallow populations [11, 29, 30, 35, 69, 95].

Octocoral mortality events following MHWs may result from a combination of the direct impacts of thermal stress on animal physiology and the disruption of the symbiosis between the host and its microbial community, consequently affecting the functioning and overall health of the host [5, 56, 96]. Corals indeed form a meta-organism called holobiont, which comprises the animal host and its associated microorganisms (i.e., protists, fungi, bacteria, archaea, and viruses) that can provide a wide range of benefits. These latter include nutritional provisioning and recycling [44, 48, 75], tolerance to environmental stress [67] and protection from pathogens by producing antibiotic compounds [41, 57, 77]. Mediterranean octocorals harbor a specific microbiota that is stable across seasons, geographic locations, and depths. For gorgonians (*Paramuricea* and *Eunicella* genera), bacteria from the *Endozoicomonas* genus dominate the microbiota (representing up to 96% of the bacterial community; [3, 99]), while bacteria from the *Spirochaetaceae* family are the main bacterial symbionts associated with the red coral *C. rubrum* (representing up to 70% of the bacterial community; [98, 99]).

Disturbances such as heat stress can induce changes in the octocoral microbiota by exerting a downward pressure on the survival of beneficial symbionts, thereby affecting the stress tolerance of the host, and promoting temperature-dependent bacterial diseases. However, there is currently limited research available on the response of the microbiome of Mediterranean octocorals to temperature anomalies representative of MHWs, and none of these studies can explain the extended mortality and tissue loss of octocorals observed during the 2022 MHWs [13, 90, 91]. They reported different responses depending on the octocoral species, sampling depth and colony, with no clear relationship between tissue loss, thermal stress intensity, and microbiome changes. In the laboratory experiments of Tignat-Perrier et al. [90, 91], decreases in the relative abundance of the major symbionts (*Endozoicomonas* or *Spirochaetaceae*) following heat stress (24 °C for several weeks) were associated with tissue loss in *Paramuricea clavata*, but not in *Eunicella cavolini* or *Corallium rubrum*. These findings were partially consistent with in situ data from gorgonian and red coral colonies during the 2011 MHWs [13], where it was observed that deep colonies of *C. rubrum* (70 m) exposed to heat stress also had a lower relative abundance of the main symbiont *Spirochaetaceae*. However, such changes in the bacterial community were not observed in shallow *C. rubrum* populations (30 m), despite their exposure to greater increases in sea temperature [13]. Altogether, these observations suggest that further studies are needed to explore changes in the microbiome of temperate octocorals following MHWs, with a broader investigation that includes multiple locations, species, and health states of octocorals. Because changes in the relative abundance of key symbionts cannot explain changes in holobiont health, other measurements, including absolute abundance of symbionts and putative pathogens, may help to better understand the symbiont/pathogen relationships.

To address the above gaps, we investigated how the microbiota associated with the gorgonian *P. clavata* and the red coral *C. rubrum* responded to the 2022 MHWs, some of the warmest heatwaves ever experienced by octocoral species in the Mediterranean Sea. To this end, we conducted an extensive sampling of ninety-six colonies of two key octocoral species, with different visual health states (0% and 90% of tissue loss) from 15 to 35 m depth. Samples were collected in two marine protected areas in the Western Mediterranean Sea, the Calanques National Park (France) and the Portofino Marine Protected Area (Italy) where MAFs are protected from most commercial and recreational activities. These areas experienced different MHW intensities, with extreme temperatures reaching 27 °C and 26 °C at 15 m depth [15], in the Calanques National Park and the Portofino Marine Protected Area, respectively. By assessing the composition of the bacterial communities associated with the octocorals using amplicon sequencing and taxon-specific qPCR analyses of the *16S rRNA* gene, we aimed to characterize actual changes in the octocoral microbiota in relation to the visual health status, collection site and depth of the colonies. This study provides important information on how octocoral-associated microbiota respond to thermal stress as well as how the bacterial symbionts contribute to the octocoral’s health. The results obtained will enable better conservation and protection of Mediterranean MAFs.

## Methods

### Study areas, temperature regimes and impacts on octocorals

**Fig. 1:**
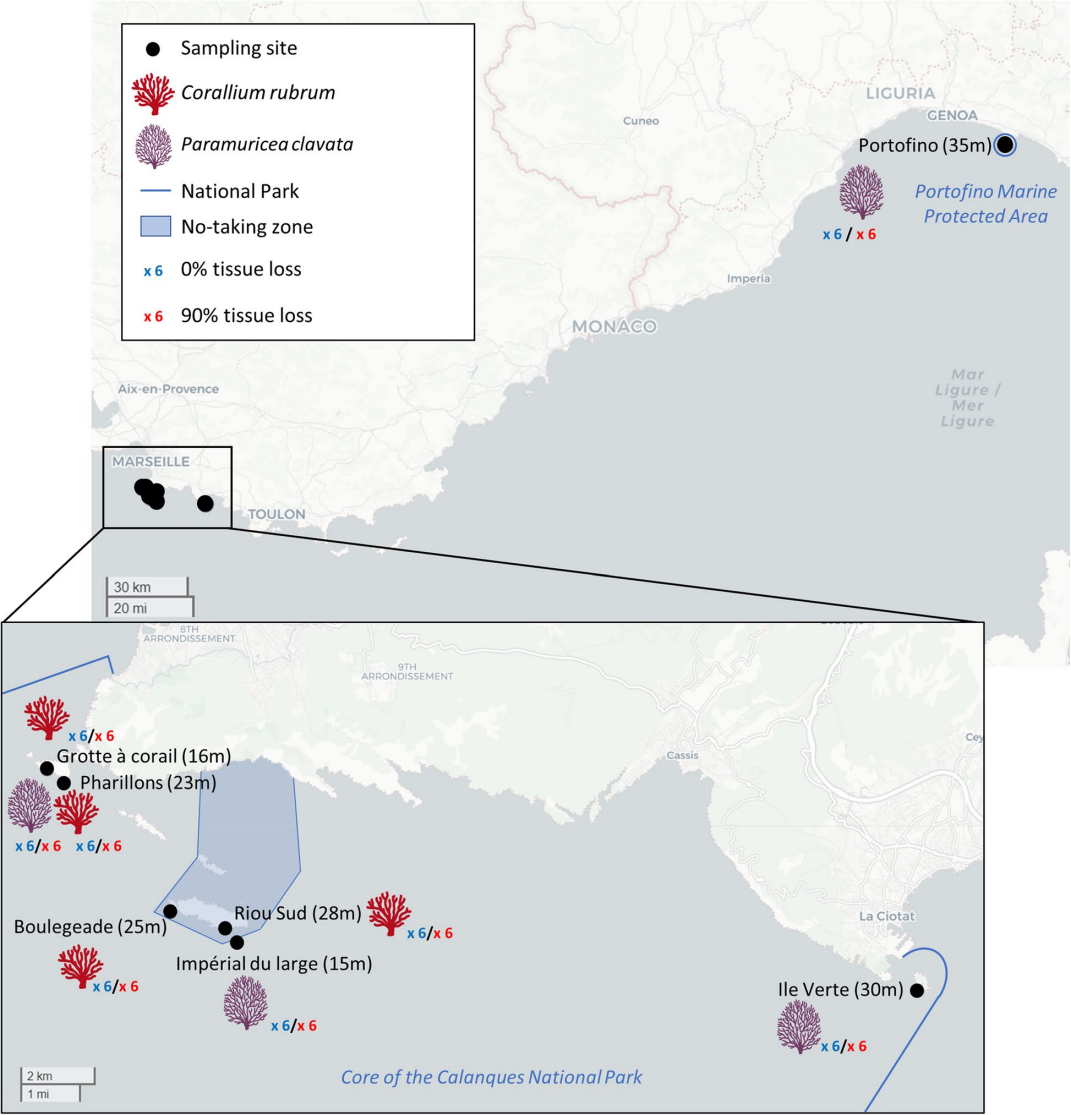
Species and number of samples collected at the different geographical locations. Collection depths (in brackets) and the number of samples per condition (blue for 0% tissue loss and red for 90% tissue loss) are indicated next to the names of the sites

Octocoral samples were collected at different locations in two marine national parks of the Northern Mediterranean Sea during the 2022 MHWs (Fig. 1). The first sampling campaign was conducted in the core area of the Calanques National Park (Marseille, France; 43 500 ha surface area) between August 31 and September 3, 2022, with the consent of the Calanques National Park (France). Among the six sampling sites, two were located in a *no-taking zone* where commercial and recreational navigation is prohibited (total surface area of the *no-taking zones* in the park: 4 634 ha). The second sampling campaign was conducted on September 5, 2022, in the Portofino Marine Protected Area (Portofino MPA, Italy) where commercial and recreational activities are controlled. Only *P. clavata* was collected in the Portofino MPA. Considering the relatively small size of the latter (346 ha surface area), coral sampling was conducted at only one site (Fig. 1) with the consent of the Portofino MPA. The collection depth ranged from 15 to 35 m depending on the collection site (Fig. 1).

**Fig. 2:**
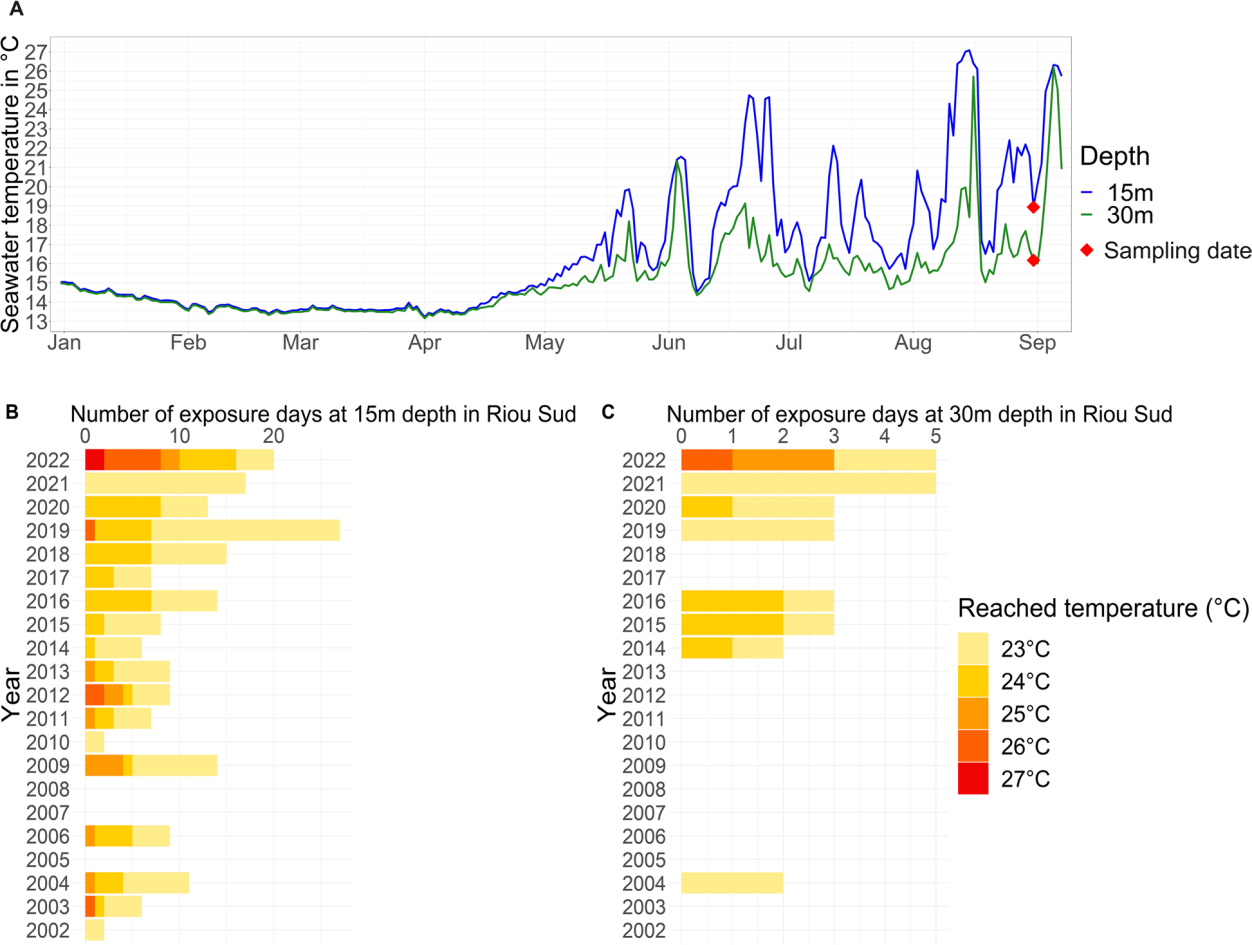
Temperature regime in 2022 and in the past 20 years at one of the collection sites (*Riou Sud*, Calanques National Park, Marseille, France). Seawater temperature at 15 m and 30 m depth from January to September 2022 (**A**), and number of exposure days at temperatures between 23 °C and 27 °C at 15 m (**B**) and 30 m depth (**C**) over the past 20 years

In 2022, the Mediterranean Sea experienced record sea surface temperatures from May to September, although temperature regimes varied by location during the MHWs. Hourly measurements of seawater temperature at *Riou Sud* (Calanques National Park, France) over the past two decades, and at *Portofino* (MPA, Italy) from 2019 to 2022 have been extracted from the T-MEDNet database (www.t-mednet.org) (Fig. 2 and Fig. S1). In the Northwestern Mediterranean Sea, thermal anomalies are either characterized by short periods (a few days) at temperatures reaching 27 °C or by long periods (several days and weeks) at warm and stable temperatures of 23–24 °C in shallow waters [15, 23]. We thus chose to graph temperatures ≥ 23 °C on Fig. 2 and Fig. S1 as 23 °C starts to be an abnormally warm temperature for the region if largely observed during the summer. During the 2022 MHWs, the seawater temperature at *Riou Sud* exhibited unprecedented records, with measurements reaching 27 °C and 26 °C at 15 m and 30 m depth, respectively (Fig. 2A). The temperature regime is characterized by upwelling events of dense and cold water coming from the deep, that occurred periodically during the MHWs (Fig. 2A). These events, that occur due to strong North-West winds, can lower seawater temperatures by up to 10 °C within a few hours before rising again [54, 58] (Fig. 2A). In 2022, *Riou Sud* experienced an exceptional frequency of days with temperatures exceeding 24 °C at both depths (17 and 3 days at 15 m and 30 m depth, respectively), surpassing any previous observations recorded in the past two decades (Fig. 2BC).

In *Portofino* (MPA, Italy), temperatures reached 26 °C and 21 °C at 15 m and 30 m depth, respectively (Fig. S1A), which correspond to 1 °C and 5 °C difference compared to those observed in *Riou Sud* (Calanques National Park, France) at the same depths (Fig. 1A). In *Portofino*, the number of days where abnormally warm temperatures (> 24 °C) were observed was not greater than the previous 3 years (Fig. 1B), suggesting that the 2022 MHWs did not exhibit unprecedented records at this location. Furthermore, at the sampling depth (35 m), the temperature only reached 19.8 °C, which is comparable to what has been observed in the three previous years (Fig. S1).

**Fig. 3:**
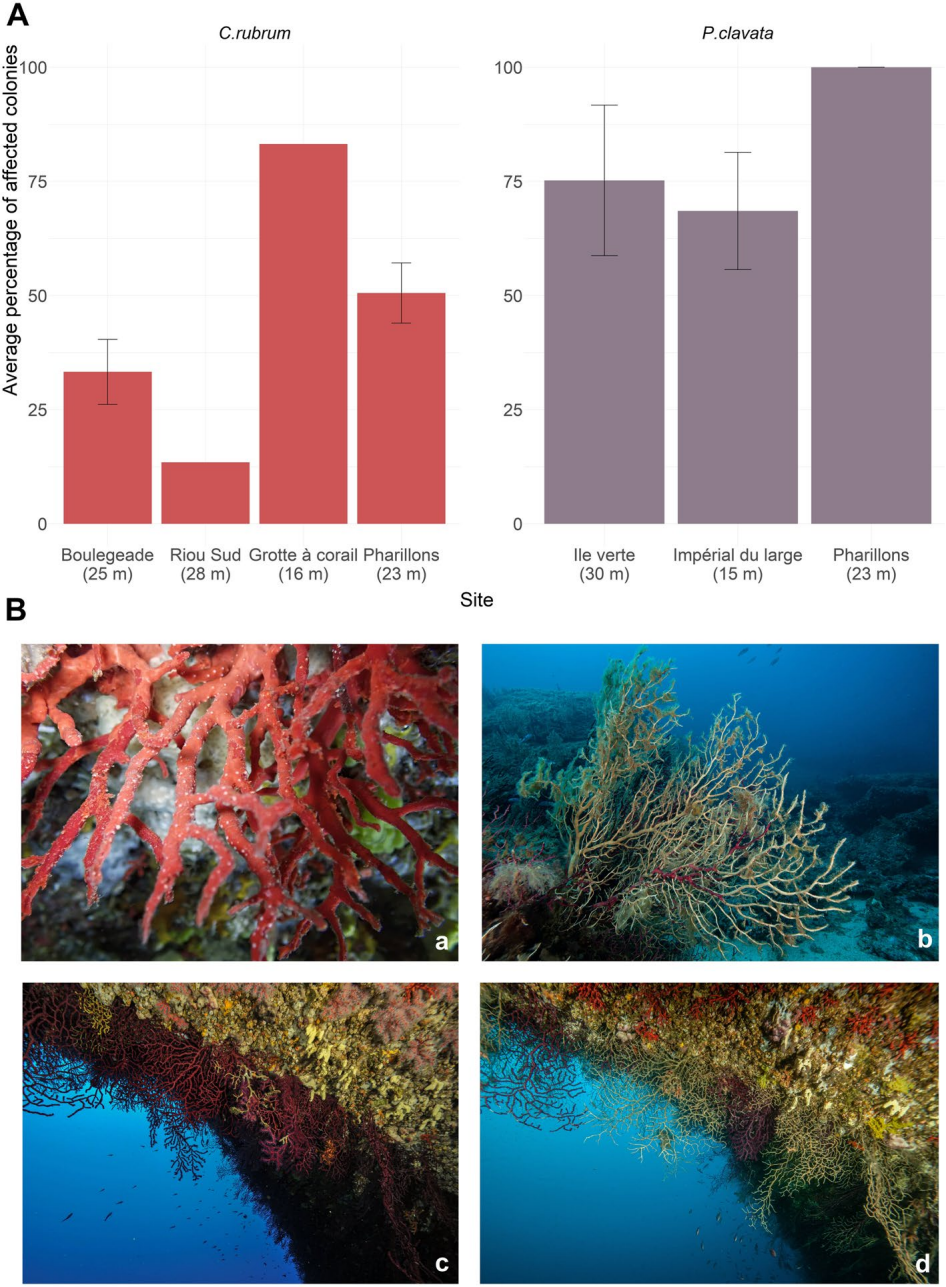
Impact of the 2022 heatwaves on coralligenous reefs of the Calanques National Park (Marseille, France). **A** Average percentage of *P. clavata* and *C. rubrum* colonies affected by tissue loss at the different sampling sites. **B** Colonies of *C. rubrum* (a) and *P. clavata* (b) exhibiting tissue loss (August 2022); Coralligenous assemblage before (c) and during (d) the heatwaves. Credit: *Septentrion Environnement*

A parallel survey of octocoral mortality was conducted to assess the percentage of colonies affected by the 2022 MHWs at the different sites in the Calanques National Park. The survey was performed according to a widely used method based on quantification of the proportion of affected colonies [29, 30] and described in detail in Estaque et al. [23]. A colony was considered affected if tissue loss represented at least 10% of the colony surface area. One or more dives were conducted at each site to enumerate the affected colonies among a hundred colonies (Table S1). Overall, *C. rubrum* suffered less from the 2022 MHWs than *P. clavata* (45% and 81% of colonies affected on average for *C. rubrum* and *P. clavata*, respectively), and octocoral populations were affected differently among sites (Fig. 3A and Table S1). Colonies of *C. rubrum* collected in *Grotte à corail* (83% of affected colonies) and *Pharillons* (51% of affected colonies) were the most affected, while *P. clavata* populations in *Pharillons* have suffered the most from the heatwaves with 100% of affected colonies (Fig. 3A).

### Sample collection

A total of ninety-six samples were collected from colonies of the red coral *C. rubrum* and the gorgonian *P. clavata* with different health status based on visible tissue loss (Figs. 1 and 3B). Samples of 3 cm were taken from colonies that showed no sign of tissue loss (0% of tissue loss) and from colonies showing 90% of tissue loss based on colony surface area. In the last case, the remaining tissue was sampled. While both *C. rubrum* (six samples per condition and site) and *P. clavata* (six samples per condition and site) colonies were sampled in the Calanques National Park, only *P. clavata* was collected in the Portofino MPA (six samples per condition; Fig. 1). Immediately after collection, samples were placed in separate Ziploc® plastic bags containing natural seawater. On board, they were transferred to Eppendorf tubes, fast-frozen in a dry shipper containing liquid nitrogen (~ 190 °C, ©CX100 Worthington Industries) and then stored at − 80 °C in the lab until further processing.

### DNA extraction

DNA was extracted using ~ 1.5 cm of octocoral sample and the DNeasy PowerBiofilm kit (QIAGEN, Hilden, Germany) with the following modifications: during the cell lysis step, 2 μL of Proteinase K (600 U/ml) was added to the sample and incubated at 60 °C for 2 h, followed by 2 min of bead beating using the CryoMill (Retch, Germany) at a frequency of 30 Hz and at room temperature. DNA concentration was measured using a Qubit fluorometer and DNA was stored at − 20 °C.

### MiSeq amplicon sequencing of the *16S rRNA* gene

#### Library preparation and sequencing

Extracted DNA samples were sent to STAB VIDA (Portugal) for a paired-end sequencing (2 × 300 bp, 600 cycles) with the V3 chemistry on Illumina MiSeq platform. Amplicon library preparation was conducted using Illumina’s standard “16S Metagenomic Sequencing Library Preparation” protocol [37]. The V3-V4 region (~ 550 bp) of the *16S rRNA* gene was amplified using the forward primer 341F 5’-CCTACGGGNGGCWGCAG-3’ and the reverse primer 785R 5’-GACTACHVGGGTATCTAATCC-3’. The fastq files containing the raw sequencing data have been deposited in the NCBI’s Short Read Archive (SRA) under the BioProject accession number PRJNA991959.

#### Bioinformatics data processing

The *16S rRNA* gene amplicon data were processed using the DADA2 pipeline (version 1.16; https://benjjneb.github.io/dada2/index.html; [8]. The sequencing of ten *P. clavata* samples and one *C. rubrum* sample was cancelled as no amplification was detected during the library preparation (amplicon PCR). On the 85 remaining samples, sequencing resulted in 14,363,708 reads ranging from 42,720 to 264,000 reads per sample (Table S2). Before any further analysis, read quality profiles were inspected and reads were filtered and trimmed with the following settings: truncLen = c(290, 250), maxN = 0, maxEE = c(2, 2). Primer sequences were also trimmed at the 5′-end of each read (17 bp and 21 bp on the reads R1 and R2, respectively). Error rates were computed and used for sequence inference. An amplicon sequence variant (ASV) table was constructed based on de-noised and merged R1 and R2 reads. Sequences smaller than 390 bp and longer than 450 bp were removed. Chimeras were detected and removed from the table using the removeBimeraDenovo function. The number of reads and sequences passing the different steps of the pipeline per sample is presented in Table S3. Taxonomy was assigned to the 12 509 ASVs using the assignTaxonomy function and the SILVA SSU reference database (version 138.1). The ASV table, the sequences of each ASV and the metadata are available as Tables S4, S5 and S6, respectively.

#### Analysis of *16S rRNA* gene sequencing data

All statistical analyses were conducted in the R environment (version 4.2.1) using the R-package *phyloseq* [50]. Observed species richness and evenness (Shannon index) were calculated using the R-package *vegan* [22]. Given the fact that rarefaction curves mostly reached the plateau (Fig. S2) and the current debate concerning the applicability of unrarefied versus rarefied data [51, 101], we conducted our alpha diversity analyses on the unrarefied ASV count table. Potential differences in observed richness were explained by constructing a generalized linear model (GLM) using a negative binomial distribution and considering site and tissue loss condition as fixed factors without taking into account the interaction as its effect was not significant (formula = Observed richness ~ Condition + Site; Table S7 ). Post-hoc tests were performed to identify potential pairwise differences (R-package *emmeans* [45]). Compositional data analysis (CoDA) [31, 74] was used to investigate the changes in the composition of the microbiota of *C. rubrum* and *P. clavata*. The raw ASV abundances were centered log-ratios (clr) transformed using the R-package *compositions* [4], after imputing zero counts based on Bayesian multiplicative replacement (Bayes-LaPlace BM method of the cmultRepl function in the R-package *zCompositions*; [63]). An Aitchison distance matrix was generated by computing Euclidean distance between samples using the clr-transformed data table. Principal component analyses (PCA), permutational multivariate analysis of variance (using functions adonis(); R-package *vegan*) were conducted to evaluate differences in the bacterial community structure between sites and conditions based on the percentage of tissue loss for each octocoral species (formula = Aitchison matrix ~ Condition + Site; Table S8). Potential pairwise differences were then considered (using the function permanova_pairwise(); R-package *ecole* [86]) adjusting the *p*-values with the false discovery rate correction (p.adjust( function; R-package stats; Table S8. Differential abundance analyses were performed to identify ASVs that were differentially abundant between health conditions (0% versus 90% of tissue loss and collection sites when the number of samples was sufficient using the R-package *ANCOM-BC* ([46]; ancombc2() function using prv_cut = 0.30 and alpha = 0.05; version 02–2023; Table S9).

### Quantitative PCR (qPCR)

Comparative qPCR was used to track changes in the abundance of total bacteria as well as the abundance of bacteria belonging to the *Spirochaetaceae* family, *Vibrio* genus and *Endozoicomonas* genus in octocoral cells. Four primer pairs targeting the *16S rRNA* gene were used including universal primers (total bacteria; [26]) and primers specific to the *Spirochaetaceae* group [36], *Vibrio* genus [89] and *Endozoicomonas* genus (modified from [83]) (Table S10). To test the specificity of the taxon-specific primers on our octocoral samples, the amplification products obtained from four samples (two per octocoral species) were sequenced using the Sanger technology (Eurofins genomics, Germany) and aligned on the *nr* database using the *blastn* algorithm. For primer pairs targeting a sequence in the V3-V4 region of the *16S rRNA* gene, primer sequences were aligned on all the ASV sequences annotated as the targeted taxon (58 ASV for *Endozoicomonas* and 190 ASV for *Vibrio* using Clustal Omega (default parameters; [84]) and sequence complementarity was verified (Tables S11 and S12). Two pairs of primers targeting the *beta actin* gene of *C. rubrum* and *P. clavata* were designed using Primer3 (http://primer3.sourceforge.net/) and the host genomes, as internal controls (Table S10).

For all primer pairs, efficiency was evaluated using different primer concentrations and doing an amplification of a series of tenfold dilutions of a mix of all DNA samples as template. The specificity of the product was assessed from a melting curve program. The amplification results were plotted as C_t_ vs. log_10_[DNA] and the amplification efficiencies were calculated using formula *E* = 10^(1/slope)^ [68]. Primer concentrations and efficiencies for each targeted gene are given in Table S10.

qPCRs were carried out on all samples in duplicate on a QuantStudio3 qPCR machine (Applied Biosystems). Amplification reactions contained 0.4 µL of each primer (concentrations in Table S7), 10 µL of SensiFAST SYBR master mix (ThermoFisher) and 2 µL of diluted DNA (1/20) in a final volume of 20 µL. Amplification was done using standard cycling conditions, i.e., 95 °C for 10 min, followed by 40 cycles of 95 °C for 15 s and 60 °C for 1 min. The relative quantification of each selected gene was determined in comparison to the *beta* actin reference gene based on the Pfaffl method [68]. Fold changes (or relative expression ratio) of the different genes in the 90% tissue loss condition were calculated and expressed in comparison to the 0% tissue loss condition.

Two-way non-parametric Kruskal–Wallis analyses were performed to test differences in the total concentration of bacteria and the abundance of *Spirochaetaceae*, *Vibrio* and *Endozoicomonas* bacteria per host cell between the health conditions and the sampling sites for each octocoral species (Table S13).

## Results

### Bacterial richness associated with octocorals with different tissue loss conditions (0 and 90% tissue loss)

**Fig. 4:**
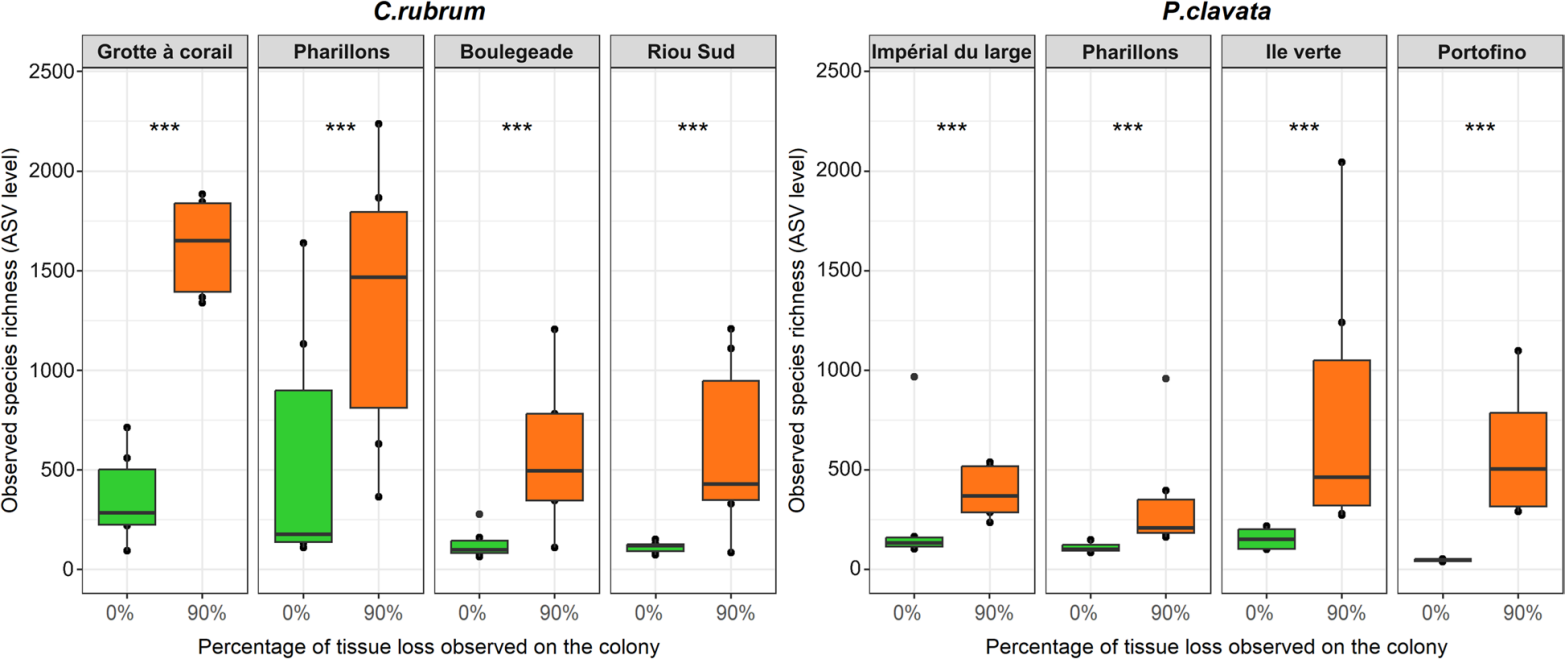
Estimation of the bacterial species richness in *C. rubrum* (left) and *P. clavata* (right) depending on the collection site and the colony health status (0% and 90% tissue loss). Statistical significance levels of differences between octocorals experiencing tissue loss or not are indicated by stars (*** for *p* < 0.001)

In colonies with 0% tissue loss, the bacterial species richness was site-specific in *C. rubrum*, with *Grotte à corail* and *Pharillons* showing higher richness values (average observed richness of 361 and 562 ASVs, respectively) compared to *Boulegeade* and *Riou Sud* (average observed richness of 129 ASVs and 113 ASVs, respectively; (post-hoc tests 0.0001 < *p* < 0.004, Fig. 4 and Table S7)). No difference was observed between sites in *P. clavata* (post-hoc tests 0.46 < *p* < 1, Fig. 4 and Table S7). Species richness increased by 4 and 3.3 times on average in colonies of *C. rubrum* and *P. clavata* with 90% tissue loss compared to colonies with no tissue loss (Estimate Std. = 1.4; Error = 0.19; z-value = 7.3; *p* = 2.9 × 10^–13^ and Estimate Std. = 1.3; Error = 0.23; z-value = 5.9; *p* = 3.01 × 10^–9^ for *C. rubrum* and *P. clavata*, respectively; Fig. 4 and Table S7). Consequently, colonies with 90% tissue loss showed higher species richness in *Grotte à corail* and *Pharillons* compared to *Boulegeade* and *Riou Sud* for *C. rubrum* (post-hoc tests 0.0001 < *p* < 0.004), while no difference between sites was observed for *P. clavata* (post-hoc tests 0.38 < *p* < 1, Fig. 4 and Table S7).

### Changes in the structure of the bacterial communities associated with octocorals with 90% tissue loss

#### Differential abundance analyses based on compositional data

**Fig. 5:**
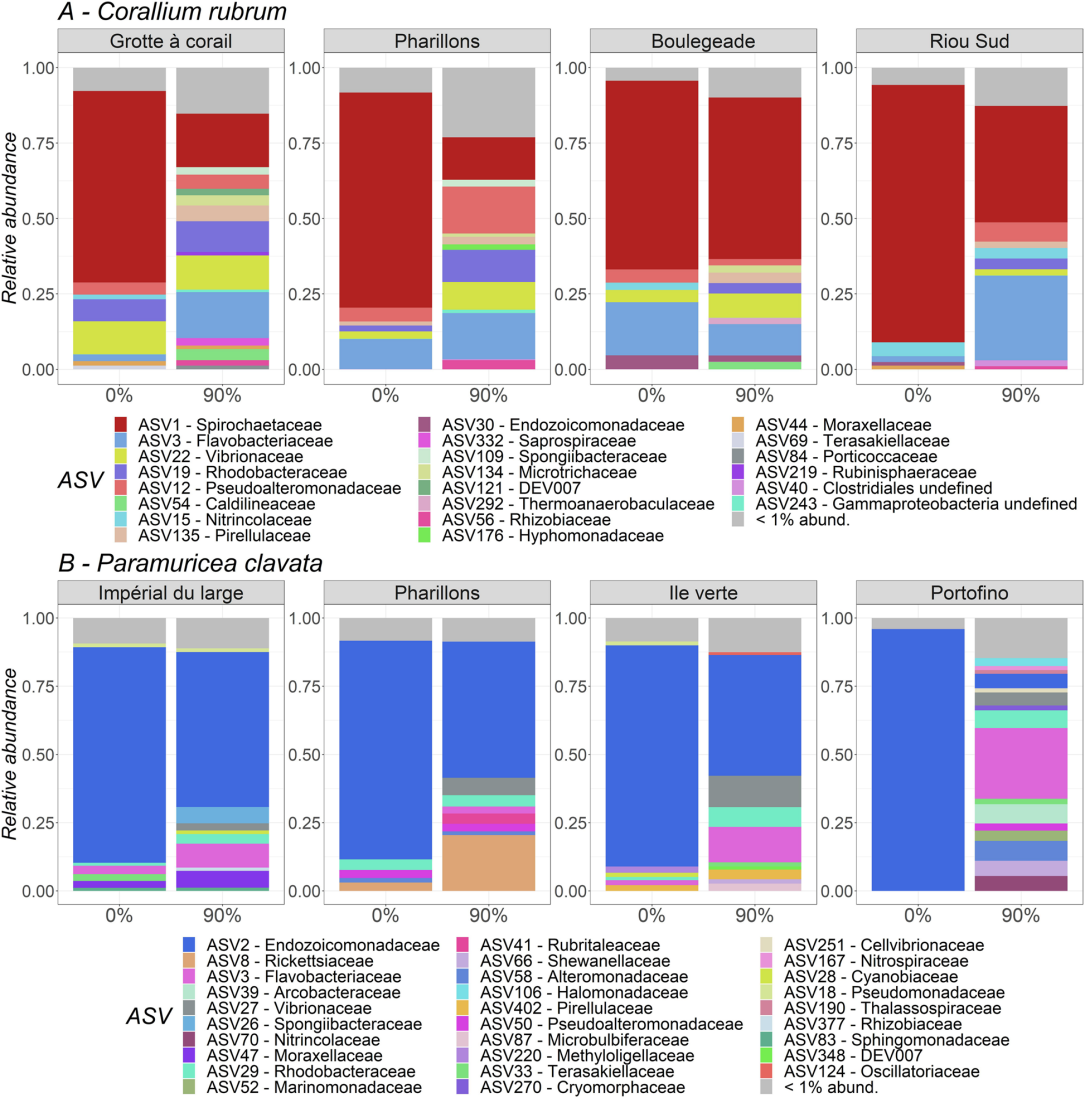
Relative abundance of the ASVs associated with *C. rubrum* (**A**) and *P. clavata* (**B**) depending on the collection site and the percentage of tissue loss (0% and 90%)

Overall, the bacterial communities associated with the colonies with 0% tissue loss were dominated by ASV1- *Spirochaetaceae* (72%), ASV5- *Spirochaetaceae* (11%) and ASV7- *Flavobacteriaceae* (6.1%) in *C. rubrum*, and by three *Endozoicomonadaceae*, ASV2, ASV6 and ASV4 (78%, 9% and 4% on average, respectively) in *P. clavata* (Figs. 5 and S4). For *C. rubrum*, no site-specific differences were observed in the structure of the bacterial communities associated with 0% tissue loss colonies (post-hoc tests 0.18 < *p* < 0.62; Figs. 5A, S3, S4 and Table S8). In *P. clavata*, the structure of the bacterial communities associated with 0% tissue loss colonies collected in *Portofino* and *Ile verte* were significantly different from the other sites (post-hoc tests 0.02 < *p* < 0.03, Figs. 5B, S3, S4 and Table S8).

For both species, overall, the structure of the bacterial communities significantly differed between the two health (tissue loss) conditions (*F* = 3.2; d.f. = 1; *p* = 1 × 10^–4^ and *F* = 1.5; d.f. = 1; *p* = 0.002 for *C. rubrum* and *P. clavata*, respectively) (Fig. 5 and Table S8).

**Fig. 6:**
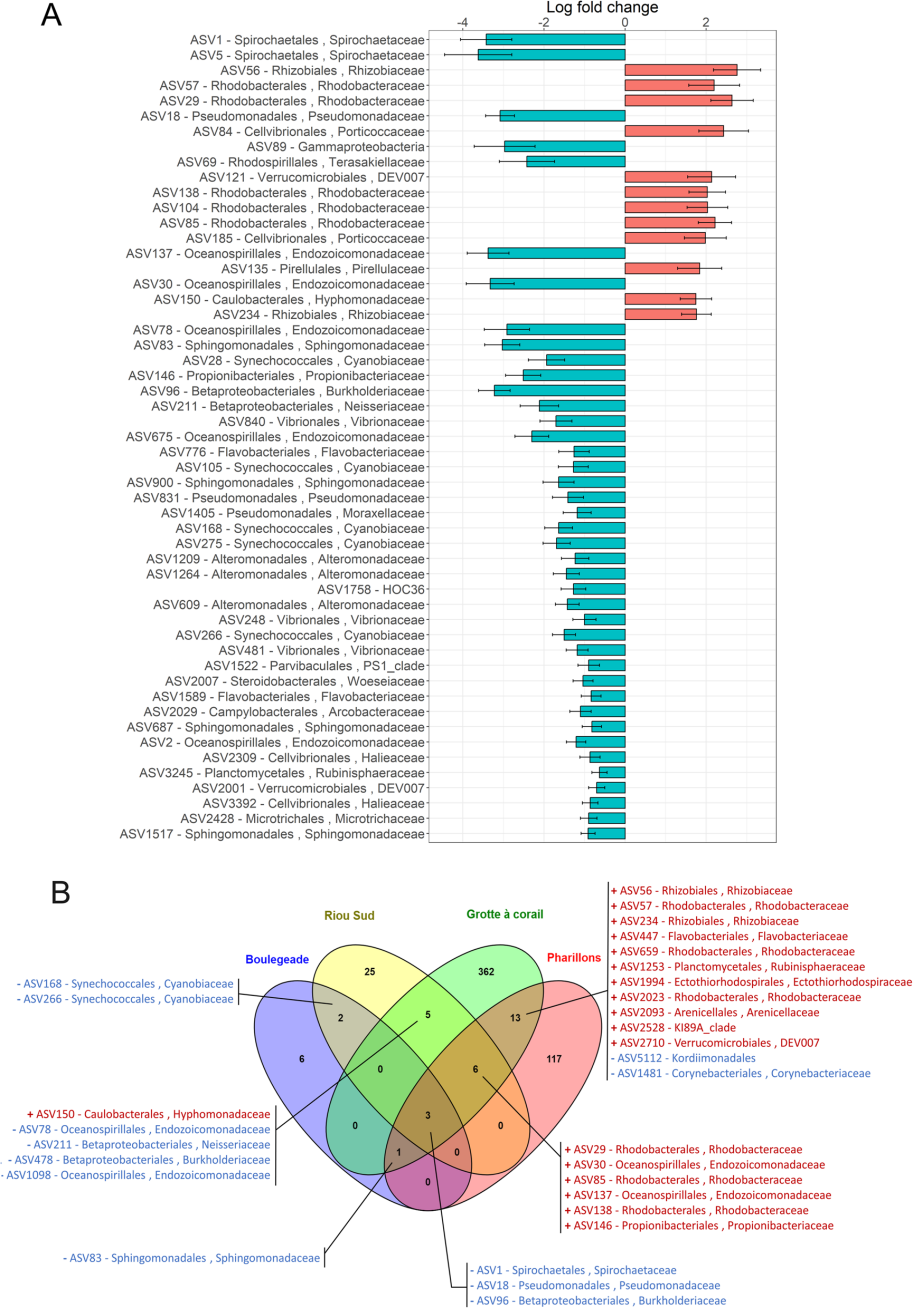
Bacterial ASVs differentially abundant between *C. rubrum* colonies with 0% and 90% tissue loss. **A** Difference in relative abundance calculated as log fold change (natural log) in the 90% tissue loss condition compared to the 0% tissue loss condition, all sites combined. **B** Venn diagram of the bacterial ASVs differentially abundant between tissue loss percentages and common between sites (‘ + ’ in red and ‘-’ in blue indicating a significant increase or decrease of the ASV in the 90% tissue loss condition, respectively)

Additionally, the bacterial communities associated with *C. rubrum* colonies showing 90% tissue loss displayed distinct structural patterns between all sites (post-hoc tests 0.02 < *p* < 0.05; Table S8), except between *Pharillons* and *Boulegeade* (post-hoc test *p* = 0.11; Table S8). Considering all sites together, 53 ASVs were differentially abundant, with 41 ASVs less abundant and 12 ASVs more abundant in the 90% tissue loss condition compared to the 0% tissue loss condition (Fig. 6). Among the dominant ASVs that significantly decreased, the ASV1- *Spirochaetaceae* and ASV5- *Spirochaetaceae* decreased by two to three times in the colonies with 90% tissue loss (ASV1: average relative abundance of 60% and 25% in the 0% and 90% tissue loss condition, respectively, and ASV5: average relative abundance of 10% and 3% in the 0% and 90% tissue loss condition, respectively; Figs. 5 and 6A). The ASVs whose abundance increased in the samples with 90% tissue loss were composed of 42% of *Rhodobacteraceae*, including ASV29 and ASV57 which were 39 times more abundant on average (average relative abundance of 0.015% and 0.36% in colonies with 0% and 90% tissue loss, respectively; Figs. 5 and 6A). When the differential analysis is conducted by site, *Grotte à corail* and *Pharillons* showed a larger number of differentially abundant ASVs between tissue loss conditions (390 and 140 ASVs, respectively) compared to *Riou Sud* and *Boulegeade* (41 and 12 ASVs, respectively; Fig. 6B). Moreover, around 15% of the differentially abundant ASVs in *Grotte à corail* and *Pharillons* belonged to the *Rhodobacteraceae* family, while this percentage dropped to 0.09% in *Riou Sud* and 0% in *Boulegeade*.

**Fig. 7:**
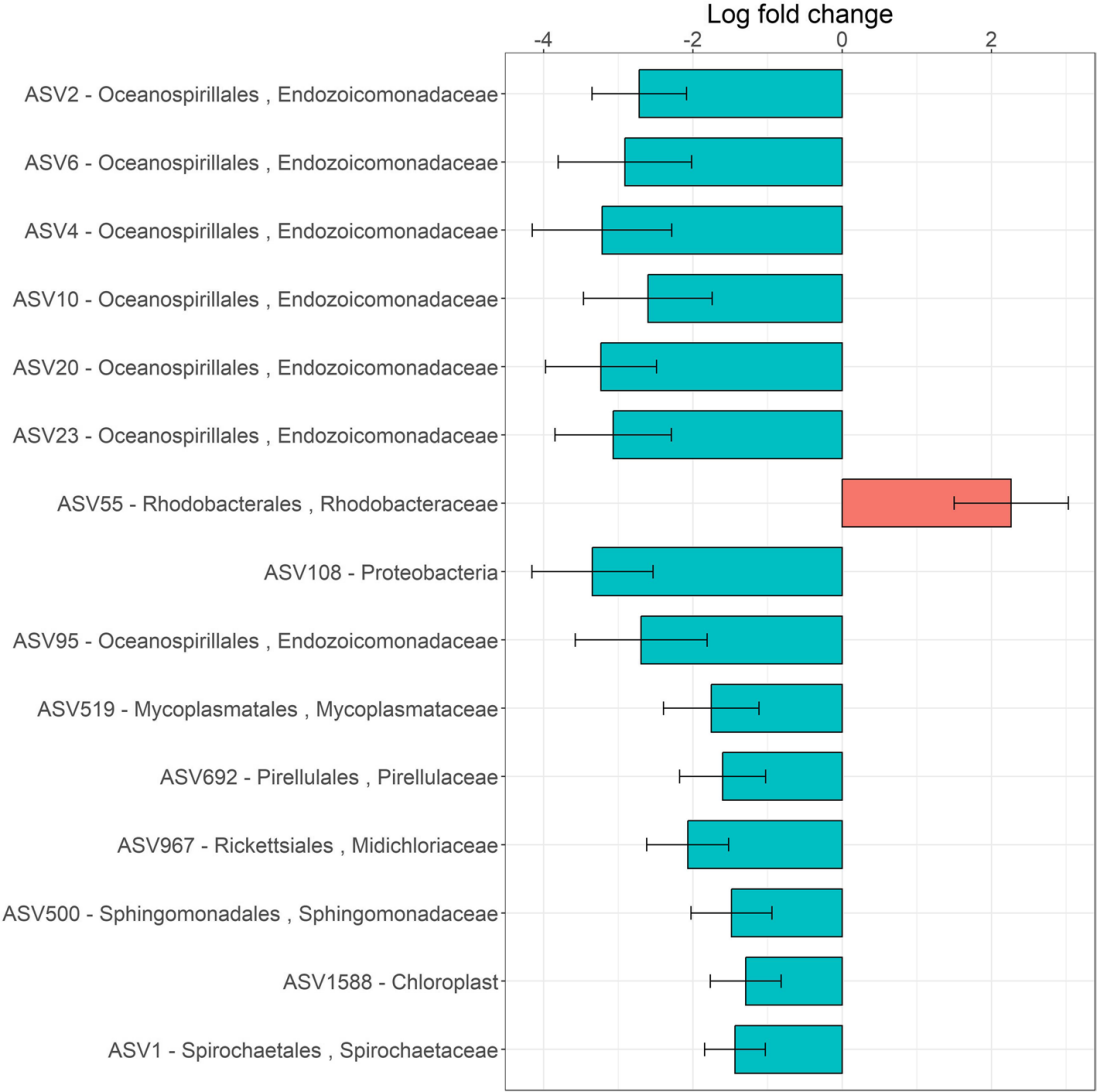
Bacterial ASVs differentially abundant in colonies of *P. clavata* with 0% and 90% tissue loss, all sites combined. The difference in relative abundance is calculated as log fold change (natural log) in the 90% compared to 0% tissue loss condition

In *P. clavata*, differential abundance analyses identified 14 ASVs, including 7 *Endozoicomonadaceae*, that were significantly less abundant in the 90% tissue loss condition compared to the 0% tissue loss condition (Fig. 7 and Table S9) The first two differentially abundant ASVs, ASV2- *Endozoicomonadaceae* and ASV6- *Endozoicomonadaceae*, both decreased by two times (ASV2: average relative abundance of 62% and 29% in colonies with 0% and 90% tissue loss, respectively; ASV6: average relative abundance of 7% and 3% in colonies with 0% and 90% of tissue loss, respectively; Figs. 5 and 7 and Table S9). Only ASV55-*Rhodobacteraceae* was found to be 44 times more abundant in *P. clavata* colonies with 90% of tissue loss compared to the colonies with 0% tissue loss (Fig. 7 and Table S9). Differential abundance analyses were not done per site due to the insufficient number of samples in certain sites and conditions (≤ four samples).

#### Comparative absolute quantification of specific bacterial taxa based on qPCR analyses

**Fig. 8:**
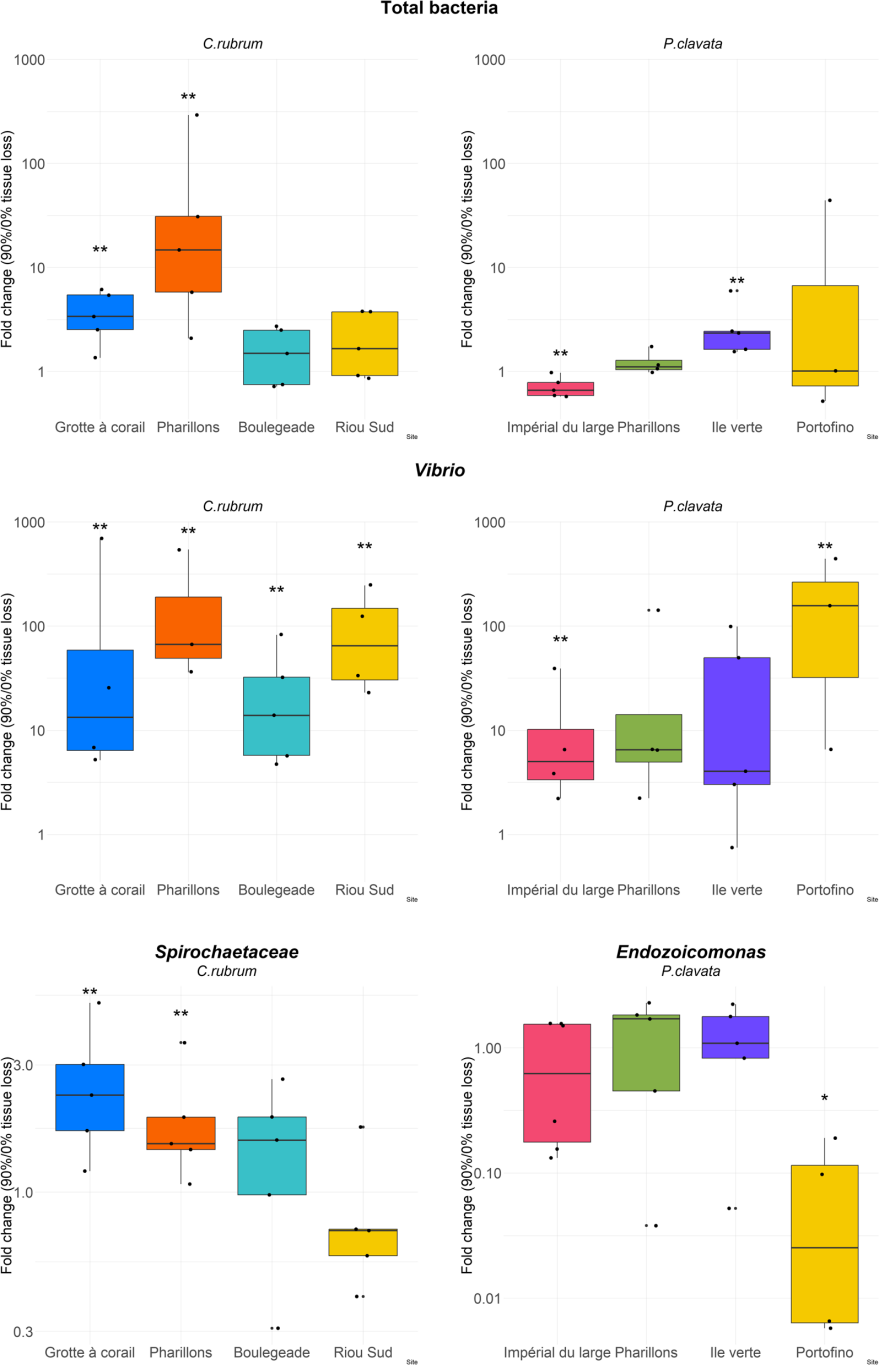
Relative expression ratio (fold change) between the 90% and 0% tissue loss conditions of the different targeted genes. Concentration per host cell of the total bacteria (**A**), *Vibrio* bacteria (**B**), *Spirochaetaceae* (**C**) and *Endozoicomonas* bacteria (**D**) per site. Graphs are presented in logarithmic scale. Statistical significance levels of differences between octocorals experiencing tissue loss or not are indicated by stars (* for *p* < 0.05 and ** for *p* < 0.01)

For *C. rubrum*, the total concentration of bacteria per host cell significantly increased in the colonies with 90% tissue loss compared to the colonies showing no tissue loss in *Grotte à corail* (*p* = 0.003; 4 times higher on average in the 90% tissue loss condition) and *Pharillons* (*p* = 0.005; 70 times higher on average in 90% tissue loss condition) (Fig. 8 and Table S13). For *P. clavata*, the total bacterial concentration only increased in the 90% tissue loss colonies collected in *Ile Verte* (*p* = 0.005; 2.8 times higher on average in the 90% tissue loss condition; Fig. 8 and Table S13).

For both octocoral species, the concentration of *Vibrio* per host cell was higher in colonies showing 90% tissue loss compared to colonies with 0% tissue loss in all sites (*p* values < 0.05; Fig. 8 and Table S13), increasing up to 313 times (*Riou Sud*) and 3126 times (*Portofino*) in *C. rubrum* and *P. clavata*, respectively, in the 90% tissue loss condition.

The concentration of *Spirochaetaceae* per host cell was higher in colonies with 90% tissue loss compared to the colonies with no tissue loss in *C. rubrum* at the sites *Grotte à corail* (*p* = 0.003; 2.7 times higher on average in the 90% tissue loss condition) and *Pharillons* (*p* = 0.005; 2 times higher on average in the 90% tissue loss condition) (Fig. 8 and Table S13).

Only in colonies of *P. clavata* from *Portofino*, the concentration of *Endozoicomonas* per host cell was lower in colonies with 90% tissue loss compared to the colonies with no tissue loss (*p* = 0.01; 14.3 times lower on average in the 90% tissue loss condition) (Fig. 8 and Table S13).

## Discussion

The aim of this study was to get a clearer picture of the impact of MHWs on MAFs, particularly on the intricate relationship between the tissue loss of Mediterranean octocorals and the observed changes in their associated bacterial microbiome. Therefore, we investigated changes in the microbiota of octocorals following record-breaking MHWs that hit the Western Mediterranean Sea in summer 2022 [32, 39, 73], affecting up to 80% of the colonies of the red coral *C. rubrum* and 100% of the colonies of the gorgonian *P. clavata* depending on the site. In this study, we collected samples of *C. rubrum* and *P. clavata* with either 0% or 90% tissue loss at different sites. Using taxon-specific qPCR analyses, we demonstrated that the absolute abundance of the dominant symbionts (*Spirochaetacae* and *Endozoicomonas* in *C. rubrum* and *P. clavata*, respectively) did not change with thermal stress, suggesting that colony tissue loss was not associated with a diminution of their abundance in the microbiome. However, the relative abundance of these symbionts decreased in corals with 90% tissue loss due to a significant increase in the total abundance of other bacteria, including putative pathogens such as *Vibrio* and *Rhodobacterales*. Taken together, our study suggests that octocoral tissue loss during MHWs is due to the combination of heat stress and overgrowth of putative pathogenic bacteria. Finally, we found a site- and species-specific response of the octocoral microbiome to heat stress. While *C. rubrum* was more affected at the shallowest (and warmest) depths, the greatest changes in the microbiome of *P. clavata* were not related to temperature alone. These findings contribute to our understanding of the intricate relationships between host organisms, their microbiota and environmental stressors, and provide valuable insights into the impact of MHWs on MAFs in the Mediterranean Sea.

### The 2022 Mediterranean heatwaves: the worst ever recorded

An analysis of seawater temperature records in *Riou Sud* (Marseille) at 15 and 30 m depths shows that the summer of 2022 was the warmest in the last 20 years. While temperatures above 24 °C had never been measured at 30 m depth before 2022 and only on a maximum of 4 days per year at 15 m depth (in 2009 and 2012), the year 2022 showed 3 and 10 days with temperatures above 24 °C at 30 and 15 m depth, respectively, indicating that the water column warmed for the first time on record down to 30 m depth in Marseille. Laboratory experiments have identified a temperature of 23–24 °C as a thermal threshold for many Mediterranean octocoral species, above which tissue loss and mortality have been observed [10, 34, 72, 90, 91, 93]. Thus, after several months (from May to September 2022) of exposure to MHWs, many octocoral colonies showed impaired health and tissue loss throughout the Northwestern Mediterranean Sea [23, 32]. A significantly higher percentage of affected colonies of octocorals has been observed at shallower depths than at greater depths in the Calanques National Park (Marseille) [23], suggesting that octocoral tissue loss is closely related to the duration and magnitude of thermal stress as observed in tropical corals [1, 24, 43]. However, visual observation of the tissue showed that not all colonies were in the same state of health, as some showed 90% tissue loss or were dead, while other colonies showed 0% tissue loss. Within a population, this could be due to small-scale differences in water flow and ‘perceived’ temperature [6], food availability [25] or host genotype [40, 62]. To explore whether changes in the bacterial microbiome could explain the observed tissue loss in Mediterranean octocorals, we examined bacterial abundance variations in colonies with 0% and 90% tissue loss. Even if the observed microbiome changes could be either responses to environmental shifts or the tissue loss itself, or potentially drivers of the tissue loss, the results suggest that the microbiome may influence host resilience or recovery, ultimately contributing to an explanation for the observed tissue loss in Mediterranean octocorals.

### Colony tissue loss is not associated with a decrease in the abundance of the main bacterial symbionts

The bacterial microbiome of colonies with 0% tissue loss showed a high relative abundance of the main symbionts, comparable to non-stressed or healthy colonies, which are dominated by *Endozoicomonas* in *P. clavata* [42, 76, 99] and *Spirochaetaceae* in *C. rubrum* [98, 100], regardless of depth, season, or geographic location. The absence of tissue loss or significant changes in the microbiota suggests that these colonies were potentially healthy at the time of sampling, even if they should have suffered from heat stress, as thermal anomalies were recorded to depth of 30 m. It would have been interesting to tag these colonies and revisit them after the MHWs to assess their overall well-being. If they continued to show no tissue loss later in the season, these colonies may represent heat stress resistant genotypes that warrant in-depth characterization.

In octocoral colonies with 90% tissue loss, we observed a significant decrease in the relative abundance of the main symbionts compared to those with 0% tissue loss (47% and 38% decrease for *C. rubrum* and *P. clavata*, respectively). This is in agreement with previous observations of a decrease in the relative abundance of the dominant bacteria in the microbiome of heat-stressed corals [62, 91, 97, 102]. However, this pattern is not always associated with tissue loss, as evidenced in recent studies of [90, 91] and Corinaldesi et al. [13] on Mediterranean octocorals. This inconsistency is maybe due to the fact that the relative quantification of the symbionts does not accurately reflect actual changes occurring within the microbiota [16, 28], and the quantification of the absolute abundance of symbionts is necessary to understand microbial dynamics.

**Fig. 9:**
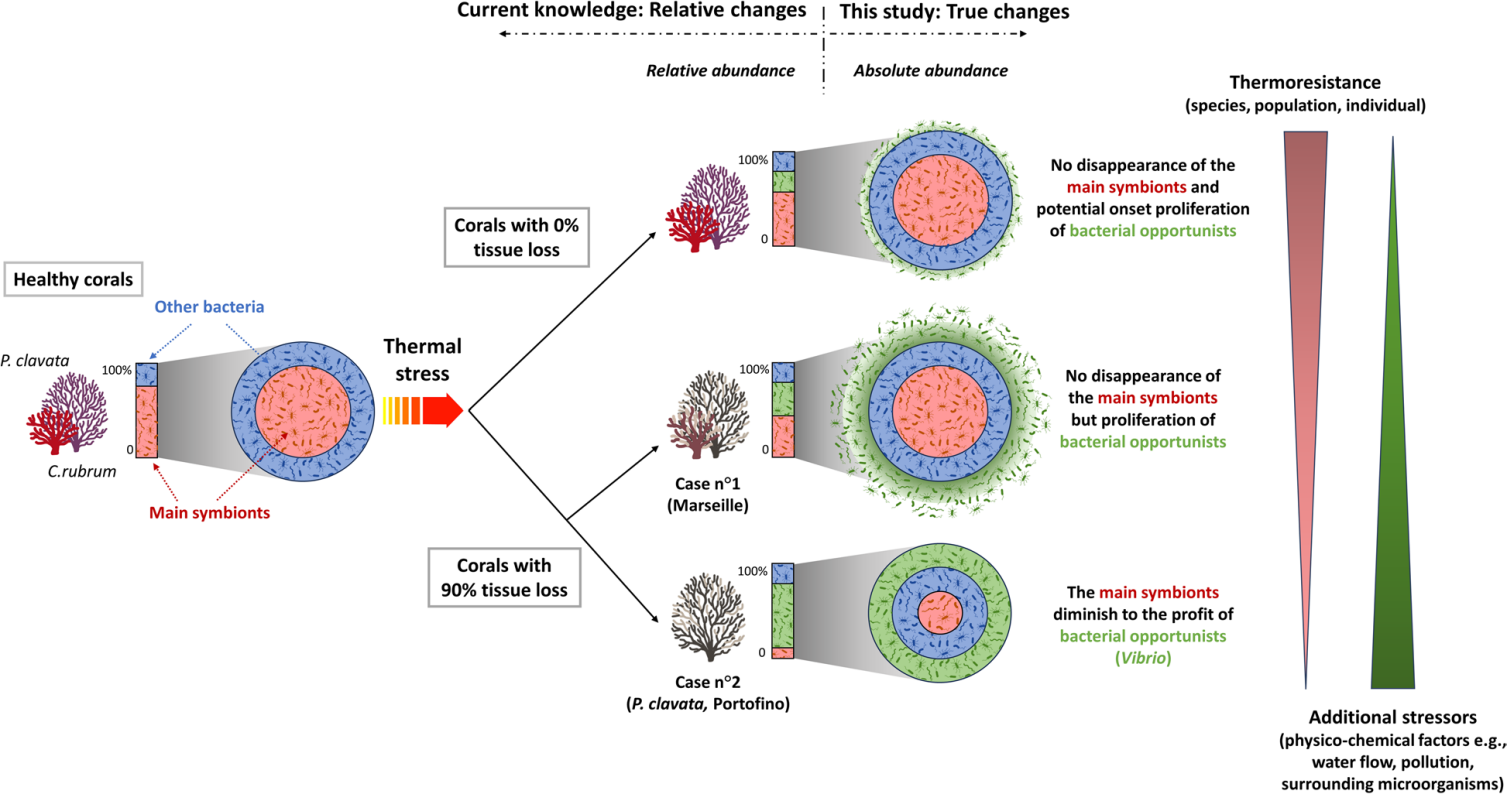
Differential responses of the octocoral microbiota to the marine heatwaves of 2022. By providing complimentary qPCR-based data to amplicon sequencing information, our study brings valuable insight on the microbiota changes occurring in two octocoral species experiencing the marine heatwaves of 2022. Biological and environmental factors were also identified as putative drivers of the differential responses

A limited number of studies have investigated the response of coral symbionts by absolute quantification of microbiota members, primarily due to the challenge of designing taxa-specific primers caused by the lack of knowledge regarding the genomes of most symbionts [70, 83]. In this study, the use of primers specific to the *16S rRNA* gene of *Spirochaetaceae* and *Endozoicomonas* revealed that colonies with 90% tissue loss did not exhibit a significant decrease in the abundance of their dominant symbionts. Instead, our study provides compelling evidence that the decline in the relative abundance of the dominant symbionts within colonies of *C. rubrum* and *P. clavata* experiencing tissue loss is, in most cases, due to the proliferation of opportunistic bacteria (saprophytic or commensal) on the octocoral tissue (Fig. 9). This pattern is largely observed in colonies sampled in Marseille. However, *P. clavata* colonies collected in *Portofino* actually showed a decrease in the absolute abundance in *Endozoicomonas* as well as the most significant changes in bacterial community composition, despite being at the deepest and coldest collection site. This suggests that *P. clavata* populations from *Portofino* might be more susceptible to heat stress, or that other factors could have altered their microbiota. For example, *Vibrio* bacteria, which has been monitored by qPCR, increased 120 times more in *P. clavata* colonies collected in *Portofino* in comparison to the ones sampled in Marseille, suggesting that the overgrowth of opportunistic bacteria could have been more important in the *Portofino* population. *P. clavata* has been identified as particularly sensible to stress-related diseases, which may explain this overgrowth of opportunistic bacteria, and the higher impact of the MHWs [2, 72, 91] on this coral species. Additionally, the Portofino Marine Protected Area is known to be exposed to various anthropogenic stressors, including water contamination and organic matter enrichment [21, 55, 64], which could have represented additional stressors for *P. clavata* colonies during MHWs. In Marseille, none of the octocoral colonies showed a decrease in the absolute abundance of the dominant symbionts after the 2022 MHWs. Changes observed in the microbiome were directly related to the thermal stress intensity. Indeed, the colonies of *C. rubrum* which displayed the most substantial changes in the composition of the bacterial communities between colonies with 0% and 90% tissue loss as well as the highest alpha diversity in both 0% and 90% tissue loss conditions, were sampled in the shallowest sampling sites (*Grotte à corail*-15 m depth and *Pharillons*-23 m depth), which were also the warmest.

The lack of substantial decrease in the absolute abundance of the dominant symbionts (*Spirochaetaceae* in *C. rubrum* and *Endozoicomonas* in *P. clavata*) in the Marseille populations suggests that there is a promising potential for these octocorals to restore their microbiota to a ‘healthy’ state after the MHWs, if their functions remain unaffected. This recovery process may occur rapidly in colonies with 0% tissue loss, or within the remaining tissue in colonies with 90% tissue loss. Future studies should consider the recovery of microbiota after MHWs to identify potential reversals, in microbiome community composition but also in their functions. Functional profiling of the microbiome (e.g., metagenomic and metatranscriptomic analyses) would allow to document changes in gene presence or expression and identify actively participating members of the microbiome.

### Octocoral tissue loss is associated with a relative increase in *Rhodobacterales* and site-specific bacterial opportunists

In colonies with 90% tissue loss, we observed a significant increase in bacterial diversity, which is likely attributed to the proliferation of opportunistic bacteria. The shift towards a more diverse bacterial community is a commonly observed phenomenon in heat-stressed organisms [20, 82, 87, 90, 91], including octocorals and may be a consequence of the temperature-induced changes in the bacterial growth dynamics and/or the octocoral loss of control over the microbiota [38, 49, 82]. Among the opportunistic bacteria, *Vibrio sp.* have been shown to frequently increase in the microbiota of heat-stressed corals [47, 91, 94]. Although the sequencing results did not reveal an increase in the relative abundance of *Vibrio sp.* in octocorals with 90% of tissue loss, we observed a significant increase in the qPCR based-absolute abundance of these bacteria (464 and 487 times increase on average in *C. rubrum* and *P. clavata*, respectively), regardless of the sampling site. The impact of the proliferation of *Vibrio sp.* on coral stress tolerance and mortality however remains largely unknown, but some *Vibrio sp*. have been shown to be pathogenic for corals [47, 88]. Additionally, we observed a higher relative abundance of *Rhodobacterales* in the microbiome of *P. clavata* and *C. rubrum* colonies with 90% of tissue loss in the Calanques National Park (Marseille). *Rhodobacterales* have a widespread distribution in various oceanic regions and are particularly prevalent as primary surface colonizers in temperate coastal waters [17]. They exhibit a capacity to thrive and multiply during phytoplankton blooms that often occur during heatwaves, as they can use newly generated organic matter by attaching themselves to marine particles [7]. Furthermore, *Rhodobacterales* have been implicated in coral diseases, including tissue loss diseases that affect stony corals [12, 27, 79, 80]. Hence, the potential relative increase of these bacteria, if confirmed to correspond to an actual increase in absolute abundance, could potentially contribute to the observed tissue loss in our colonies. However, further research is necessary to confirm this hypothesis.

Bacteria that proliferated within the microbiome of colonies exhibiting 90% tissue loss were site-dependent, suggesting that both the surrounding physicochemical conditions and the seawater microorganisms may play a role in the observed bacterial changes. It also suggests that coral tissue loss is not associated to specific pathogens. The variation in seawater bacterial communities and the prevalence of specific bacteria in the vicinity of the colonies might have influenced the diversity of opportunistic bacteria that thrived within the stressed octocoral microbiota. Indeed, no *Rhodobacterales* were observed in *Portofino* samples, although *P. clavata* colonies lost their tissue to the same extent as those sampled in Marseille. In particular, the microbiota of the *Portofino* gorgonians showing tissue loss was characterized by a large increase in the relative abundance of *Alteromonadales* and *Flavobacteriales*, a group of species associated with tissue loss in tropical corals [49, 53, 80].

## Conclusion

Our study sheds light on the complex dynamics of the coral’s microbiota in response to the 2022 MHWs, which severely affected Mediterranean octocorals down to 30 m depth. The results highlight the importance of considering both relative and absolute quantification methods when assessing the effects of thermal stress on the coral microbiome. Based on the measurements of the absolute abundance of the total bacteria and specific taxa, we showed that the abundance of the main symbionts was not affected in most colonies showing 90% tissue loss, and we suggest that octocoral tissue loss was probably the consequence of both heat stress and the proliferation of putative pathogens. Still, further studies should investigate the functional changes in the microbiota using a combination of metatranscriptomics and metagenomics, as heat stress might alter the microbiota’s functions, for example by down-regulating genes related to metabolic functions that normally benefit the host and the holobiont. The loss of the main symbiont *Endozoicomonas* in *P. clavata* population in *Portofino*, while exposed to smaller thermal anomalies, suggests that additional factors (surrounding physico-chemical factors, seawater microorganisms, host genotypes) than heat stress might have contributed to the observation of more drastic changes in the microbiota. Considering the above suggestions, future studies are urgently needed to elucidate the underlying processes that confer higher thermotolerance to some individuals and populations as the frequency and intensity of MHWs are expected to increase [85], threatening the survival of MAFs in shallow and mesophotic habitats.

### Supplementary Information

**Additional file 1: Figure S1.** Temperature regime in 2022 and in the past 20 years in Portofino. Seawater temperature at 15 m, 30 m, and 35 m depth during the 2022 heatwaves (A), and number of exposure days at temperatures between 23°C and 27°C at 15 m (B), 30 m depth (C) and 35 m depth (D) over the past 3 years. **Figure S2.** Rarefaction curves for all the samples of the two coral species. **Figure S3.** Principal component analysis of the Aitchison distance matrix based on the composition of the bacterial community (ASV level) associated with *P. clavata* (A) and *C. rubrum* (B) grouped according to the site and health state of the corals. **Figure S4.** Relative abundance of the ASVs associated with *C. rubrum* (A) and *P. clavata *(B) depending on the samples, collection site and the colony health status based on the tissue loss percentage (0% and 90%).

**Additional file 2: Table S1.** Mortality study. **Table S2.** Sequencing results. **Table S3.** DADA2 pipeline outputs. **Table S4.** ASV table. **Table S6.** Metadata. **Table S7.** Alpha diversity results and statistical analysis. **Table S8.** PERMANOVA outputs. **Table S9.** ANCOM-BC outputs. **Table S10.** qPCR primers information. **Table S13.** qPCR results and statistical analysis.

**Additional file 3: Table S5.** Fasta file containing the sequences of each ASV.

**Additional file 4: Table S11.** Fasta file containing the alignments between qPCR primers targeting *Vibrio* and ASV sequences.

**Additional file 5: Table S12.** Fasta file containing the alignments between qPCR primers targeting *Endozoicomonas* and ASV sequences.

## Data Availability

The datasets generated and analysed during the current study are available in the NCBI’s Short Read Archive (SRA) under the BioProject accession number PRJNA991959. Measurements of seawater temperature have been extracted from the public T-MEDNet database (www.t-mednet.org).

## Abbreviations

MAF: Marine Animal Forest
MHW: Marine Heat Wave
*C. rubrum*: *Corallium rubrum*
*P. clavata*: *Paramuricea clavata*
MPA: Marine Protected Area
