# Supplementary material for: Unveiling microbiome changes in Mediterranean octocorals during the 2022 marine heatwaves: quantifying key bacterial symbionts and potential pathogens
Source: Microbiome. 2023 Dec 5;11:271. doi: 10.1186/s40168-023-01711-x (PMC10696765; doi:10.1186/s40168-023-01711-x)
Supplement: Supplementary file 2 — Additional file 1: Figure S1. Temperature regime in 2022 and in the past 20 years in Portofino. Seawater temperature at 15 m, 30 m, and 35 m depth during the 2022 heatwaves (A), and number of exposure days at temperatures between 23°C and 27°C at 15 m (B), 30 m depth (C) and 35 m depth (D) over the past 3 years. Figure S2. Rarefaction curves for all the samples of the two coral species. Figure S3. Principal component analysis of the Aitchison distance matrix based on the composition of the bacterial community (ASV level) associated with P. clavata (A) and C. rubrum (B) grouped according to the site and health state of the corals. Figure S4. Relative abundance of the ASVs associated with C. rubrum (A) and P. clavata (B) depending on the samples, collection site and the colony health status based on the tissue loss percentage (0% and 90%). [file 40168_2023_1711_MOESM1_ESM.docx]

**Supplementary Information: Figures**


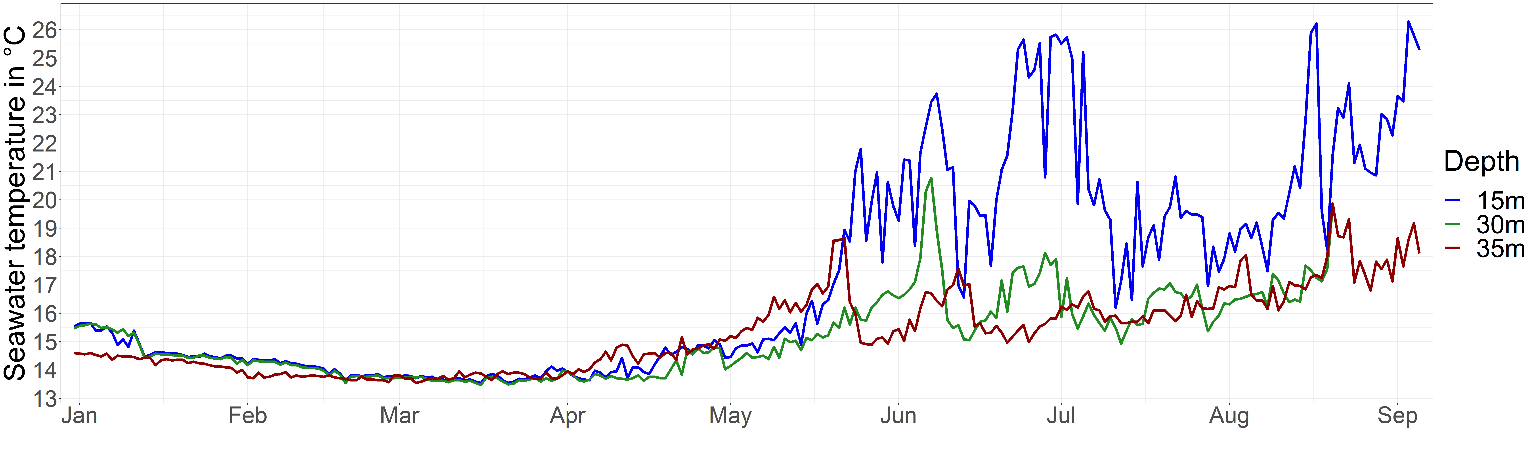

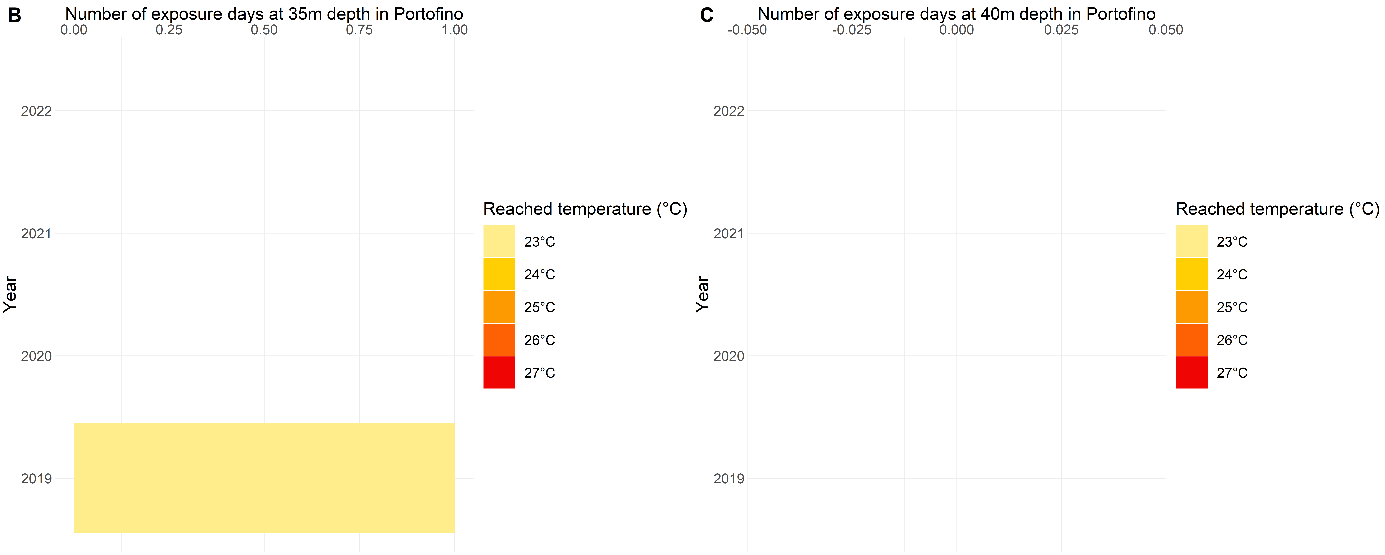

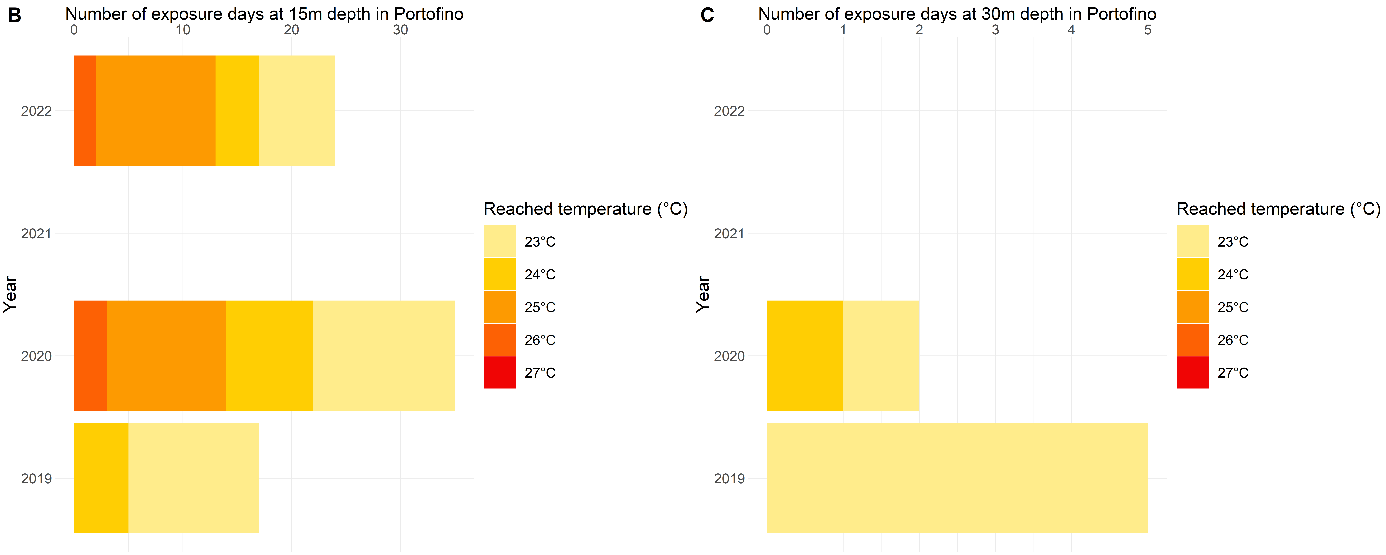

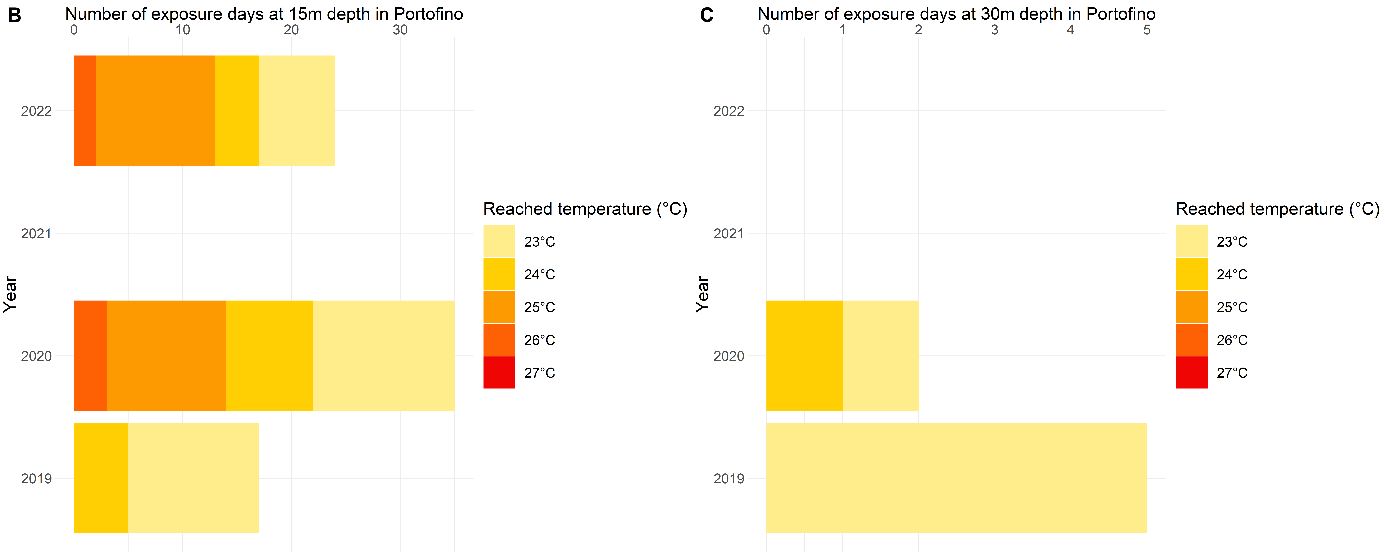

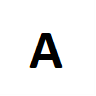

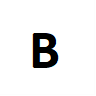

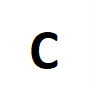

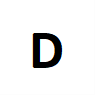


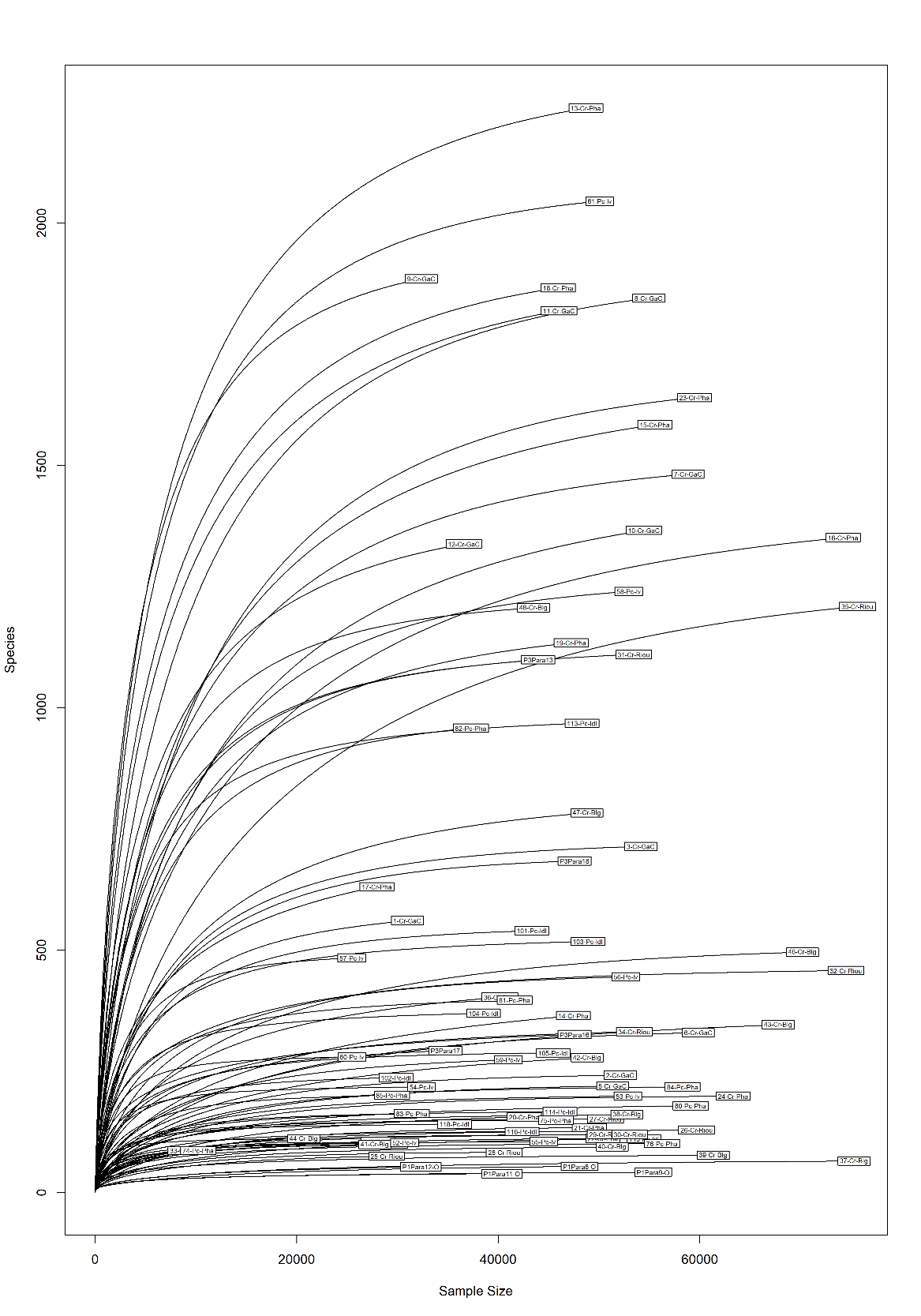
**Figure S1:** **Temperature regime in 2022 and in the past twenty years in Portofino.** Seawater temperature at 15 m, 30 m, and 35 m depth during the 2022 heatwaves (**A**), and number of exposure days at temperatures between 23°C and 27°C at 15 m (**B**), 30 m (**C**) and 35 m depth (**D**) over the past three years.

**Figure S2:** Rarefaction curves for all the samples of the two coral species.


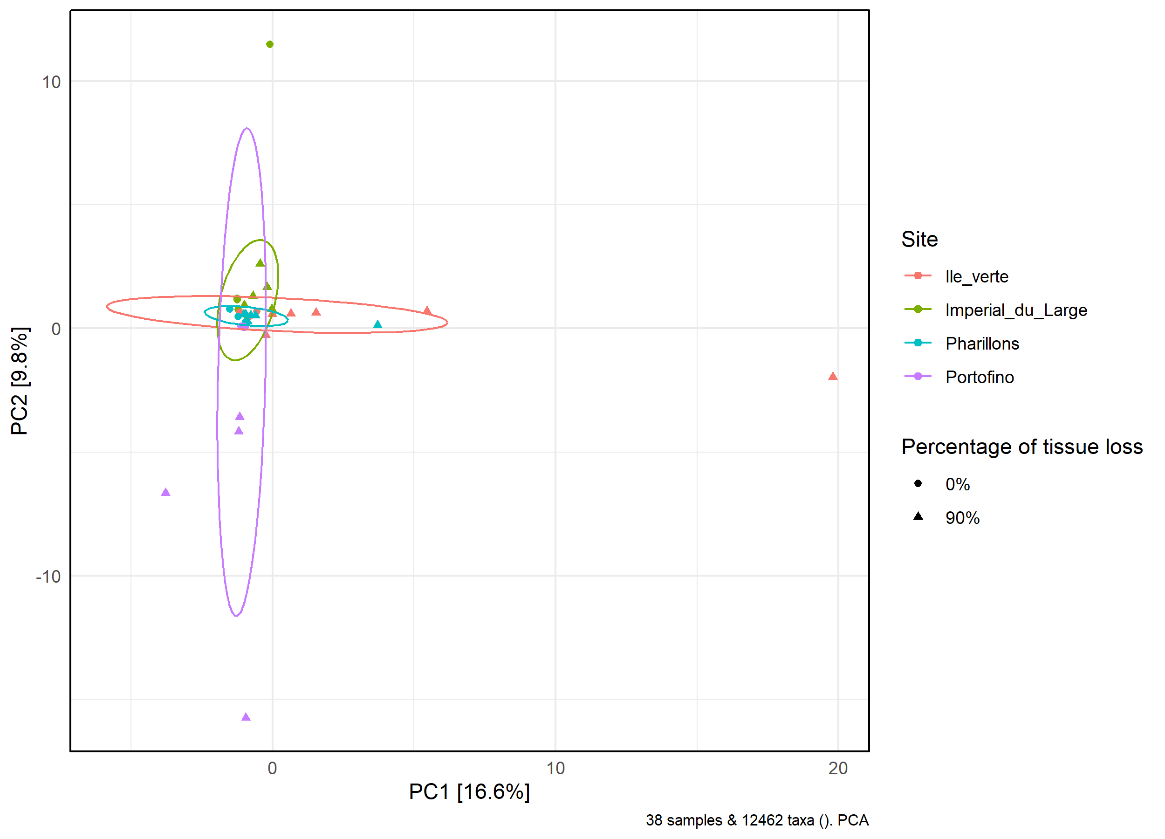

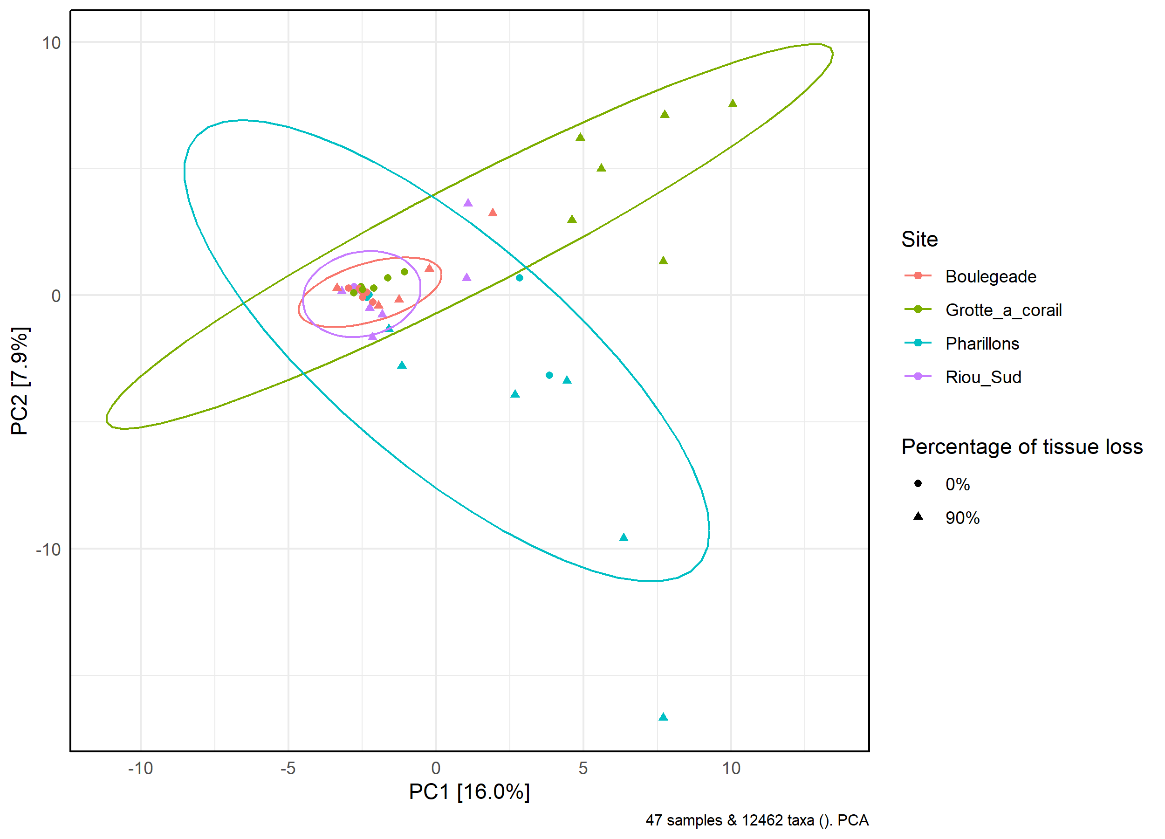

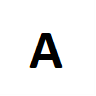

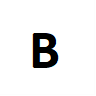


**Figure S3:** Principal component analysis of the Aitchison distance matrix based on the composition of the bacterial community (ASV level) associated with *P.clavata* (A) and *C. rubrum* (B) grouped according to the site and health state of the corals.


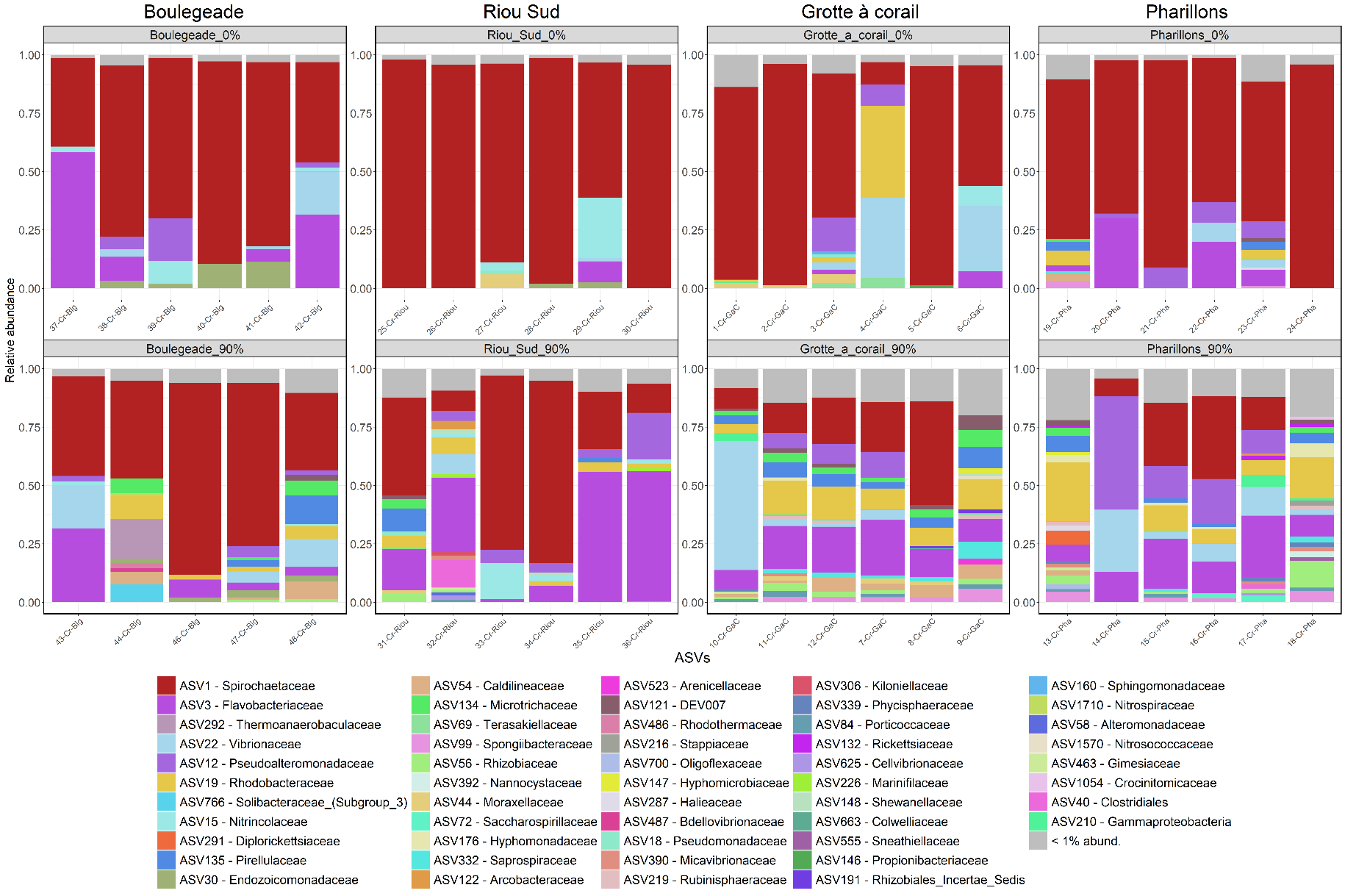

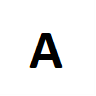

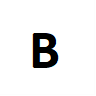

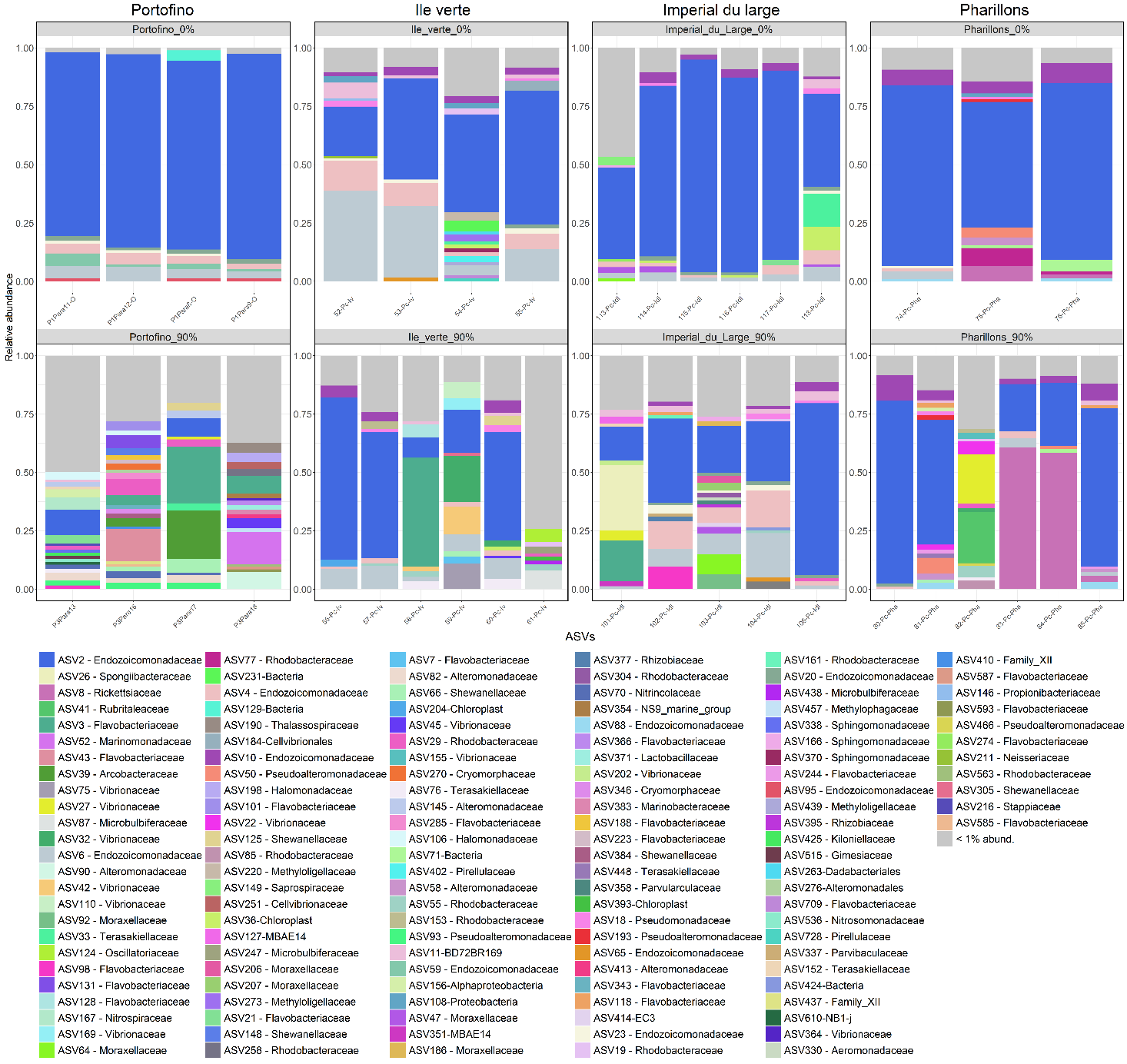


**Figure S4:** Relative abundance of all ASVs associated with *C. rubrum* (**A**) and *P. clavata* (**B**) depending on the samples, collection site and the colony health status based on the tissue loss percentage (0% and 90%).
